# Having a Post-Retirement Job: Improvisation and Containing Commitments

**DOI:** 10.1093/geroni/igab046.1604

**Published:** 2021-12-17

**Authors:** Loretta Platts, Agnieszka Ignatowicz, Hugo Westerlund, Dara Rasoal

**Affiliations:** 1 Stress Research Institute, Department of Psychology, Stockholm University, Stockholm, Stockholms Lan, Sweden; 2 University of Birmingham, Birmingham, England, United Kingdom; 3 Stockholm University, Stockholm University, Stockholms Lan, Sweden; 4 Dalarna University, Falun, Dalarnas Lan, Sweden

## Abstract

This qualitative paper focuses on individuals who work after pensionable age, a distinctive period in the late career when workers are supported by the known and reliable income of a pension. Using constant comparative analysis, we analyzed interviews from a purposive sample of 25 Swedish people in their late sixties and early seventies. We examined conditions for being in paid work in terms of enabling factors (self-employment, shift work, shortage occupation), improvisation, and the role of chance. The interviews revealed that post-retirement workers took charge of the aspects of work that mattered most to them, evading the disciplinary aspects of work by controlling scheduling and limiting the duration of their commitment. These constrained commitments had knock-on effects of improving psychosocial working conditions. Women and immigrants—groups facing low pensions—experienced the greatest financial consequences of being unable to work in their retirement years in order to supplement their pension income.

